# Fractional-Order Bioimpedance Modelling for Early Detection of Tissue Freezing in Cryogenic and Thermal Medical Applications

**DOI:** 10.3390/s26020603

**Published:** 2026-01-15

**Authors:** Noelia Vaquero-Gallardo, Herminio Martínez-García, Oliver Millán-Blasco

**Affiliations:** Department of Electronics Engineering, Eastern Barcelona School of Engineering (EEBE), Technical University of Catalonia—BarcelonaTech (UPC, Universitat Politècnica de Catalunya), E-08019 Barcelona, Spain; noelia.vaquero@upc.edu (N.V.-G.); oliver.millan@upc.edu (O.M.-B.)

**Keywords:** modelling, electrical equivalent model, fractional-order, medical applications, energy transfer, Vector Network Analyzer (VNA)

## Abstract

Cryotherapy and radiofrequency (RF) treatments modulate tissue temperature to induce therapeutic effects; however, improper application can result in thermal injury. Traditional temperature-based monitoring methods rely on multiple thermal sensors whose accuracy strongly depends on their number and spatial positioning, often failing to detect early tissue crystallization. This study introduces a fractional order bioimpedance modelling framework for the early detection of tissue freezing during cryogenic and thermal medical treatments, with the feasibility and effectiveness of this approach having been reported in our prior publications. While bioimpedance spectroscopy itself is a well-est. The corresponablished technique in biomedical engineering, its novel application to predict and identify premature freezing events provides a new pathway for safe and efficient energy-based therapies. Fractional-order models derived from the Cole family accurately reproduce the complex electrical behavior of biological tissues using fewer parameters than classical integer-order models, thus reducing both hardware requirements and computational cost. Experimental impedance data from human abdominal, gluteal, and femoral regions were modelled to extract fractional parameters that serve as sensitive indicators of phase-transition onset. The results demonstrate that the proposed approach enables real-time identification of freezing-induced electrical transitions, offering a physiologically grounded alternative to conventional temperature-based monitoring. Furthermore, the fractional order bioimpedance method exhibits high reproducibility and selectivity, and its analytical figures of merit, including the limits of detection and quantification, support its use for reliable real-time tissue monitoring and early injury detection. Overall, the proposed fractional order bioimpedance framework enhances both safety and control precision in cryogenic and thermal medical applications.

## 1. Introduction

The combination of radiofrequency and cryolipolysis offers a synergistic approach to non-invasive fat reduction. RF induces lipolysis and stimulates collagen remodeling through controlled heating, while cryolipolysis promotes adipocyte apoptosis via localized cooling. Although generally safe, improper control of thermal or cryogenic energy delivery can lead to adverse effects, including damage and sensory alterations [1,2,3].

In clinical practice, cryolipolysis treatments typically involve device-specific applicators operating at temperatures below the freezing point of adipose tissue, with treatment durations on the order of tens of minutes [4,5].

Frostbite represents a surface injury resulting from prolonged exposure to freezing temperatures, with severity ranging from mild redness and swelling to numbness, reduced blood flow, and ice crystal formation in tissues.

Precise targeting and controlled energy delivery, combined with early detection of frostbite, are essential to prevent severe tissue injury and minimize long-term sequelae [6,7,8,9,10,11,12,13,14,15]. The feasibility and effectiveness of impedance-based detection approaches have been reported in previous studies, demonstrating their ability to enable real-time identification of freezing-related electrical transitions and the onset of phase transitions [16].

During cryogenic exposure, the crystallization of intracellular and extracellular water alters its intrinsic electrical properties, offering a detectable biomarker for early-stage damage. Understanding these electrical variations is therefore fundamental for developing real-time monitoring and control strategies that improve both treatment safety and therapeutic efficacy [17,18].

Achieving accurate energy control requires precise modelling of current distribution within the heterogeneous treatment zone, which includes various tissue layers and cellular structures. Traditional equivalent circuit models based on ideal capacitors fail to adequately represent the complex electrical behavior of biological tissues, as highlighted in seminal and recent studies [17,18,19], which demonstrate the necessity of frequency-dependent and fractional-order modeling approaches for accurate tissue characterization.

In contrast, fractional-order equivalent models, particularly those based on the Cole framework, provide a more accurate and mathematically robust characterization of tissue conductance across multiple anatomical regions [12,13,19]. These models represent complex tissue using significantly fewer components than integer-order models, offering not only reduced instrumentation complexity, lower energy consumption, and minimal computational demands, but also building upon prior work that demonstrates their effectiveness in capturing tissue-specific electrical behaviour [17,20,21,22,23].

This modeling efficiency enables the implementation of reliable, real-time, closed-loop control systems for optimized energy delivery during temperature-modulated therapies.

### 1.1. Electrical Modelling of Biological Tissues

The modelling of biological tissues using electrical analogues enables the accurate characterization of their frequency-dependent impedance behavior. This approach is fundamental for understanding energy transfer in biological media and guiding the design of medical devices based on electrothermal principles. The following subsections provide a structured description of the fractional-order models employed in this study all derived from the Cole family of impedance models.

### 1.2. Objective and Rationale

The aim of this work is to develop physiologically consistent circuit models that replicate the experimental impedance spectra of human tissue while minimising structural complexity. Fractional-order models are particularly effective for this purpose, offering greater flexibility and accuracy than their integer-order counterparts by accommodating distributed relaxation behaviours and non-ideal capacitive responses [17,18,21,22,24].

Such models are especially suited for tissues with heterogeneous morphology and multilayer structure, where traditional resistor–capacitor circuits fail to provide accurate fits. Their application spans numerous fields, including bioimpedance spectroscopy [19], soil dielectric properties [21], cochlear modelling [20], muscle fatigue analysis [22], and medical thermotherapy [25,26,27].

The objectives of this study are to develop and validate a fractional-order bioimpedance modelling framework capable of detecting freezing-induced electrical transitions in adipose tissue, and to evaluate its analytical performance in terms of reproducibility, selectivity, and detection limits.

## 2. Materials and Methods

The presence of cold-induced burn blisters highlights the potential adverse effects associated with inadequate control of thermal energy during cryogenic medical or aesthetic procedures. This image is included to provide clinical context for the relevance of accurate bioimpedance-based monitoring, as such tissue damage is preceded by measurable electrical property changes that the proposed modelling framework aims to detect at an early stage, prior to the onset of irreversible injury.

**Figure 1:**
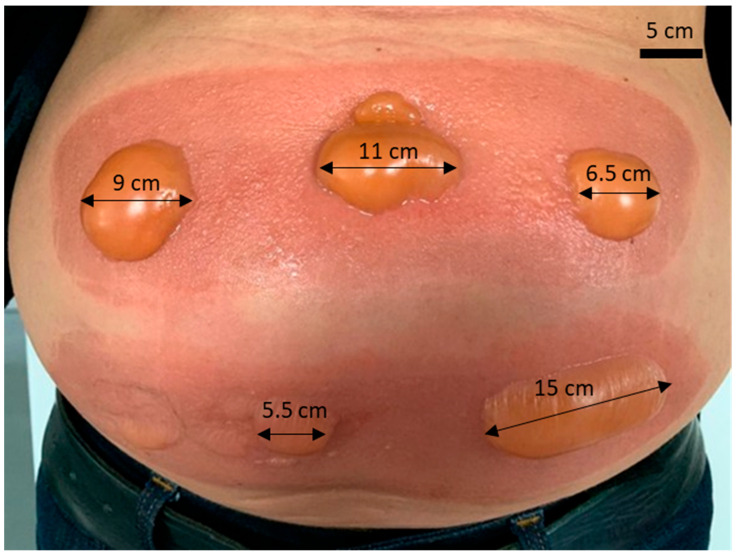
The biological response to thermal cold exposure includes erythema and skin blistering consistent with clinical presentations of frostbite Lesion dimensions are indicated directly on the image (in centimeters) to provide spatial reference (Own photograph obtained during cryogenic exposure test).

Figure 1 illustrates a clear and representative case of cutaneous injury observed in the abdominal region following excessive cryogenic exposure.

This observation highlights the importance of careful thermal energy control during cryogenic procedures. Accurate monitoring of tissue response, such as through bioimpedance measurements, can provide early warning of potential injury before irreversible damage occurs.

### 2.1. Measurement System

The passive electrical response of biomaterials over a wide frequency range was characterized by using automated vector network analysis. For this purpose, PicoVNA^®^ instruments (Pico Technology, Cambridge, UK), operating at 6 GHz and 8.5 GHz, were employed in combination with the corresponding open-source control software (v3.3). These analyzers are based on time domain reflectometry (TDR) and implement a non-destructive evaluation method [28,29]. Impedance measurements were derived from the analysis of S-parameters, which quantify the reflected and transmitted signal behavior across the device under test (DUT). The measurement configuration comprises the impedance analyzer connected to the selected skin fold area.

For impedance measurements in the lower frequency range of 0.5 to 1.5 MHz, calibration remains essential to minimize errors introduced by cables, connectors, and the measurement configuration itself. Although these factors are less critical than at higher frequencies, they can still affect both the magnitude and phase of the recorded signal. While the PicoVNA instruments are capable of operating over a wide frequency range (300 kHz to 8.5 GHz for the PicoVNA), measurements in this study were specifically conducted between 0.5 and 1.5 MHz [30,31].

The selected frequency range from 0.5 to 1.5 MHz represents a compromise between physiological sensitivity and measurement robustness. In this band, the impedance response of biological tissues is predominantly governed by the bulk conductive properties of intra- and extracellular compartments, which are directly affected by changes in water content and ionic mobility associated with thermal or cryogenic processes, including tissue freezing. At lower frequencies, electrode polarisation and interface effects may obscure tissue-related information, whereas at higher frequencies the response becomes increasingly dominated by capacitive dispersion associated with cell membranes and other fine-scale structures, which are not the primary focus of the proposed detection framework [18,30,32]. Consequently, the 0.5–1.5 MHz range provides enhanced sensitivity to freezing-induced electrical transitions while maintaining high measurement stability and reproducibility.

Additionally, operating in this lower frequency range helps minimize parasitic errors from cables, connectors, and electrodes, thereby ensuring reliable and reproducible measurements. In particular, parasitic errors refer to the undesired impedance contributions introduced by the measurement chain, including the distributed capacitance and inductance of coaxial cables, connector discontinuities, and the electrode-tissue interface. These effects, though subtle, can lead to significant deviations in impedance estimation, especially in phase-sensitive analyses. To mitigate these artefacts, a rigorous open/short/load procedure was employed, effectively relocating the reference measurement plane as close as physically possible to the tissue interface. This approach ensures that the recorded impedance values reflect the intrinsic electrical properties of the biological sample, devoid of distortions introduced by the measurement setup.

**Figure 2:**
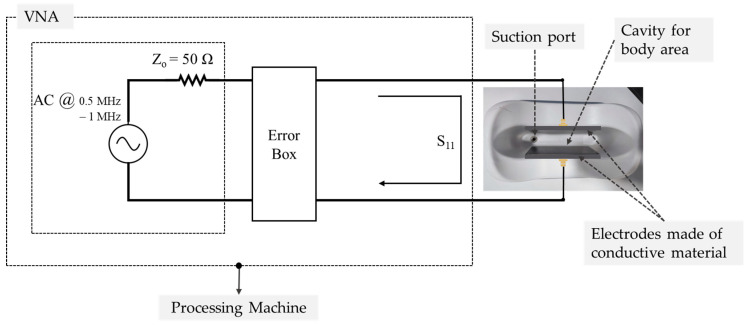
Experimental configuration for impedance measurement and data acquisition. The setup consists of a Vector Network Analyzer (VNA) interfaced with the load corresponding to the tissue region evaluated in this study. The configuration includes electrodes made of conductive material, a suction port, a cavity for body area placement, and a processing machine, as shown in the schematic representation. The same setup was used for all anatomical regions and experimental conditions.

Including this calibration reduces magnitude errors from over 1 dB to below 0.15 dB, and phase errors from over 10° to less than 2°. This represents an improvement in measurement accuracy by more than an order of magnitude. This step improves the reliability, reproducibility, and validity of the experimental result. A schematic of experimental data acquisition is represented in Figure 2.

For each selected area, all impedance measurements were performed under baseline conditions at environmental (room) temperature, prior to any cryogenic or thermal intervention, over a frequency range of 0.5 to 1.5 MHz These baseline measurements provide reference data for the fractional-order bioimpedance models, which are intended to replicate the electrical behavior of the tissue prior to the detection of impedance changes induced by cryogenic or thermal medical treatments.

Through a coaxial cable, the signal is transmitted via the emitter electrode, which is in direct contact with the tissue under evaluation. The coaxial cable shielding is connected to the receiver electrode, also in contact with the tissue under evaluation, running parallel to the emitter electrode. Both electrodes were rectangular, made of stainless steel, and had a surface area of approximately 10 cm^2^ to ensure sufficient contact and reduce edge effects. Conductive gel was applied to minimize contact impedance. The electrodes were positioned symmetrically around the region of interest, maintaining consistent inter-electrode distances for all subjects. This layout was designed to maximize reproducibility and to capture the tissue impedance of both superficial and deeper adipose layers.

The treatment-area cavity was designed to accommodate the various tissue types under investigation. Continuous contact between the plates and the tissue was ensured via the integrated suction mechanism of the device, which stabilized both the sensor and the conductive surfaces in place, thereby minimizing the potential for measurement variability due to motion or misalignment. Aluminum was selected for its favorable mechanical and electrical properties, offering stable signal transmission and enhancing overall measurement consistency.

### 2.2. Fitting Data Processing

To ensure accurate modeling, the proposed equivalent circuit must be capable of closely approximating the measured impedance spectrum, with parameter values tuned to keep deviations within a defined error margin. Each component included in the model must correspond to a physically interpretable phenomenon. The parameter optimization procedure is iterative by nature and concludes once the difference between experimental and simulated responses remains consistently within acceptable bounds.

In this study, data were analyzed and modeled using ZVIEW™ (v. 4.0h), a specialized software tool for equivalent circuit fitting that incorporates the Kramers–Kronig (KK) transform to ensure model consistency. Model accuracy was evaluated using two key statistical metrics: Root Mean Square Error (RMSE) and the coefficient of determination (R^2^) [33,34,35,36,37,38,39,40].

RMSE quantifies the absolute magnitude of the discrepancies between experimental and predicted values. It is calculated as the square root of the mean of the squared residuals. This metric is sensitive to large deviations, making it a valuable indicator of fit quality.

R^2^ represents the proportion of the variance in the experimental data that is explained by the model. Values approaching 1 denote a strong correlation and high predictive accuracy, while values near 0 indicate poor model performance. Unlike RMSE, R^2^ is dimensionless metric that provides a normalized measure of goodness-of-fit.

To ensure the robustness of the modeling approach, the Kramers–Kronig test was applied to assess linearity and causality. This involved comparing simulated Nyquist and Bode plots to the experimental spectra. Significant discrepancies may suggest structural inadequacies in the model

Following validation, electrical parameters for each tissue type were extracted by averaging five independent measurements per sample. Four different equivalent circuit models were tested for each tissue region. The optimal model, for each case, was selected based on the combination of lowest RMSE, highest R^2^, and successful compliance with the KK test, ensuring both numerical accuracy and physiological relevance in the electrical characterization.

### 2.3. Mathematical Formulation

The circuits examined in this work comprise conventional resistor and constant phase elements (CPEs). The CPE is a fractional-order impedance component that generalizes the concept of capacitance. It is characterized by two parameters: the capacitance *CPT-T* and the fractional exponent *CPE-P*. Its impedance is defined as follows [31]:

(1) $$Z_{CPE}\left(jw\right)=\frac{1}{{CPE-T\cdot \left(jw\right)}^{CPE-P}}$$

When *CPE-P*→1, the element approximates an ideal capacitor. This flexibility enables the accurate modelling of bioelectrical systems with dispersive characteristics such as those arising from complex dielectric structure of tissues, cell membranes, and intra/extracellular interactions [17,19,22,23,27,32].

The specific fractional-order circuit topologies studied are as follows:

Single-Dispersion Cole Model: This model is composed of a series resistor *R*_1_, followed by a parallel branch of R_2_ and CPE1. This configuration captures a dominant dispersion process, commonly observed in homogeneous tissues [17,22]. The mathematical model is the following:

(2) $$Z\left(jw\right)=R_{\infty }+\frac{R_{1}}{{1+\left(jw\tau \right)}^{\alpha }}$$

Double-Dispersion Cole Model: This extends the Single-Dispersion structure with an additional branch comprising *R*_3_ and CPE2, modelling secondary dispersive phenomena relevant in multilayered or anisotropic tissues such as skin or fascia [16,27]. The mathematical model is as follows:

(3) $$Z\left(jw\right)=R_{\infty }+\frac{R_{1}}{{1+\left(jw\tau_{1}\right)}^{\alpha }}+\frac{R_{2}}{{1+\left(jw\tau_{2}\right)}^{\beta }}$$

In Equations (2) and (3), Z(ω) is the complex impedance at angular frequency ω, $R_{0}$ is the resistance at zero frequency (DC resistance), and $R_{\infty }$ is the resistance at very high frequencies where capacitive effects of cell membranes and other dispersive elements are negligible. τ represents the characteristic time constant of the tissue, α(0 ≤ α ≤ 1) is the dispersion parameter describing the deviation from a purely capacitive behavior, and $j=\sqrt{-1}$ is the imaginary unit. This formulation allows separation of the purely resistive components ($R_{\infty }$) from the frequency-dependent dispersive behavior ($R_{0}-R_{\infty }$) associated with cellular and extracellular structures.

Capacitive Model Cole Model: Integrates CPE1 in series with parallel R_1_ and CPE2 combination, used to model tissues exhibiting predominantly capacitive responses [22,36].

Inductive Model Cole Model: Incorporates R_1_, a fractional inductor L_1_ and CPE1 in series. Although less common, such configurations have been reported in biological systems exhibiting inductive behavior at specific frequencies [20,23].

**Figure 3:**
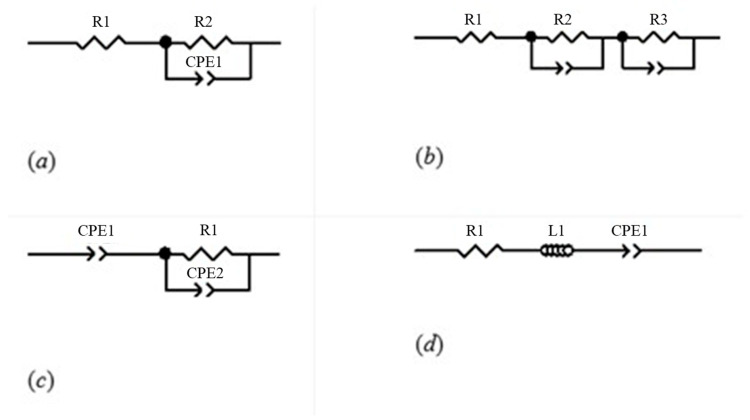
Equivalent electrical circuit representation according to the Cole model family: (**a**) Single-Dispersion Cole Model; (**b**) Double-Dispersion Cole Model; (**c**) Capacitive Model; and (**d**) Inductive Model.

These models are shown in Figure 3. They were selected not only for their ability to mimic the physical behaviour of the tissue but also for their lower computational requirement compared to traditional high-order models, facilitating implementation in embedded systems and portable instrumentation [25,35].

These schematics illustrate the different approaches for modeling tissue electrical properties. They provide a visual reference for understanding how each model captures the frequency-dependent behavior of biological tissues, which is critical for accurate bioimpedance-based monitoring.

### 2.4. Model Selection Criteria

The models described have been validated across diverse domains involving biological impedance analysis. In particular, Cole-type fractional-order circuits have demonstrated excellent agreement with experimental bio impedance data over broad frequency ranges [17,19,22,26,27,32,40,41,42].

Alternative models, such as the Debye model and the Warburg model, are not as suitable for this application. The Debye model, although effective for systems with a single relaxation process, is too simplistic to capture the multiple relaxation processes that occur in skin. Similarly, the Warburg model, which is typically used for electrochemical systems involving ion diffusion, does not account for the complex interactions seen in biological tissues. Therefore, the use of the Cole models is essential for obtaining accurate measurements, as these alternatives fail to adequately capture the variability of skin impedance across a broad frequency range.

### 2.5. Evaluation of Reproducibility, Selectivity, and Detection Limits of CPE-P Parameters

Analytical figures of merit were assessed for the extracted CPE-P parameters to evaluate the performance of the proposed bioimpedance-based detection method. Reproducibility was determined using 35 repeated measurements per anatomical region, calculating the mean, standard deviation (SD), and Average Coefficient of Variation (CVav%). Selectivity was assessed by comparing the mean values of each parameter across different anatomical regions and verifying that inter-regional differences exceeded baseline variability. Limits of detection (LOD) and quantification (LOQ) were estimated following standard analytical definitions as LOD = 3σ/S and LOQ = 10σ/S, where σ is the baseline SD (abdomen) and S is the minimum inter-regional sensitivity. Calibration plots were generated for each parameter, showing the agreement between predicted values from the model and observed measurements, allowing a visual assessment of reproducibility, selectivity, and detection capability.

## 3. Results and Discussion

This section is divided into the following: Input Data for Fitting, Electrical Equivalent Fitting, and finally, Goodness of Fit Evaluation. The measurements were evaluated using 35 observations, each corresponding to impedance data collected from the abdomen, buttocks, and thigh of individual subjects. The subjects belonged to multiple categories according to the BMI (Body Mass Index) scale: Underweight (8 subjects), Normal weight (9 subjects), Overweight (9 subjects), and Obesity (9 subjects). The results presented below correspond to the averaged values per subject, regardless of BMI category and anatomical region.

### 3.1. Input Data for Fitting

**Figure 4:**
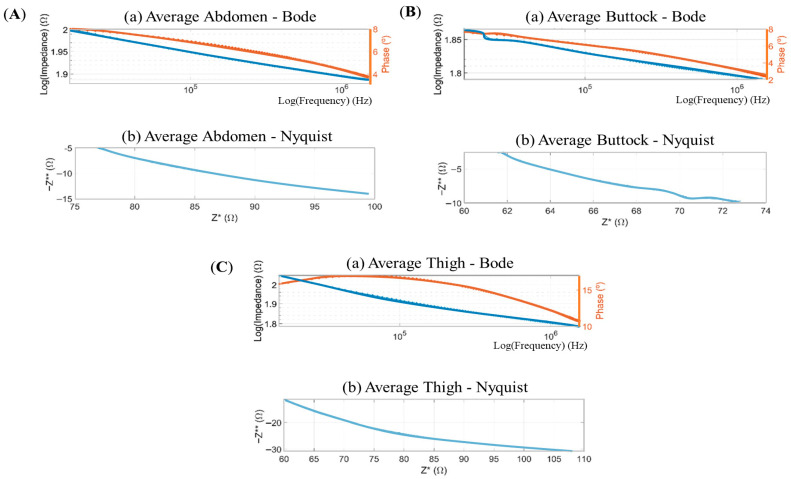
Bode and Nyquist diagrams. Upper section: Volumetric impedance plotted on a logarithmic scale (left axis) alongside phase angle (right axis) against frequency, also in logarithmic scale, ranging from 0.5 MHz to 1.5 MHz. Lower section: Negative imaginary component of impedance (−Z**) against real component of impedance (Z*). Panels show the following: (**A**) average data of abdomen of subject—Bode and average data of abdomen of subject—Nyquist; (**B**) average data of buttock of subject—Bode and average data of buttock of subject—Nyquist; and (**C**) Average data of thigh of subject—Bode and average data of thigh of subject—Nyquist.

Impedance data for the abdominal, gluteal, and femoral regions were acquired through a frequency sweep spanning 0.5 MHz to 1.5 MHz. The corresponding Bode and Nyquist representations are shown in Figure 4, respectively, for each anatomical site.

The Bode and Nyquist plots shown in Figure 4 reveal a first-order system with a predominantly capacitive phase response across all samples. In the Nyquist representation, the imaginary component of the impedance consistently exceeds the real component, with a magnitude approximately ten-times greater in all cases.

### 3.2. Electrical Equivalent Fitting

**Figure 5:**
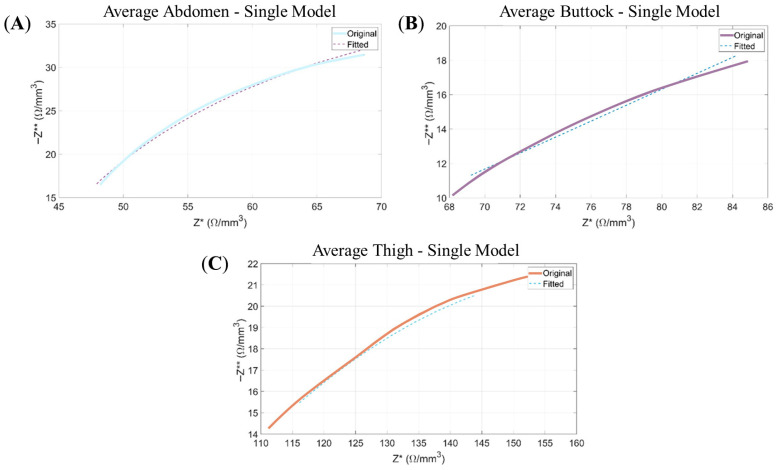
Nyquist plots: Representation of the negative imaginary component of the impedance (−Z**) as a function of its real component (Z*). The continuous line corresponds to the experimental data, while the dashed line illustrates the modelled response obtained from the Single-Dispersion Equivalent Electrical Model. Panels show the following: (**A**) the average data of abdomen for the Single Model; (**B**) the average data of buttock for Single Model; and (**C**) the average data of thigh for Single Model.

The adjusted data corresponds to the Single-Dispersion Model and the Double-Dispersion Model. For both models, Nyquist plots comparing the fitted data to the original experimental measurements are provided, along with the respective adjusted electrical parameter values. To avoid an excessive extension of graphical content, results for the capacitive and inductive models are omitted due to their inadequate fitting performance. This conclusion is substantiated by the goodness-of-fit metrics (Figure 7c,d). For the Single-Dispersion Model, the results are presented from Figure 5 and its electrical parameter values in Table 1.

As illustrated in Figure 5, the Single-Dispersion Model exhibits a satisfactory fit to the experimental data, as reflected in the agreement observed in the Nyquist plots and the fulfillment of the Kramers–Kronig (KK) relations.

Table 1 presents the numerical values of the equivalent circuit elements. These electrical parameters correspond to the mean values derived from fitting the experimental impedance data obtained from four distinct subjects per anatomical region, following the application of the selected modelling approach.

Table 1 illustrates that the CPE1-P parameters approximate a value of 1, signifying that the constant phase element (CPE) functions analogously to a capacitor.

This CPE-P values close to unity have been reported for adipose and skeletal muscle tissues under baseline conditions, reflecting predominantly capacitive responses associated with cellular membrane integrity and extracellular fluid distribution [17,30].

Regarding the Double-Dispersion Model, its results are depicted in Figure 7, with the corresponding electrical parameter values detailed in Table 2.

**Figure 6:**
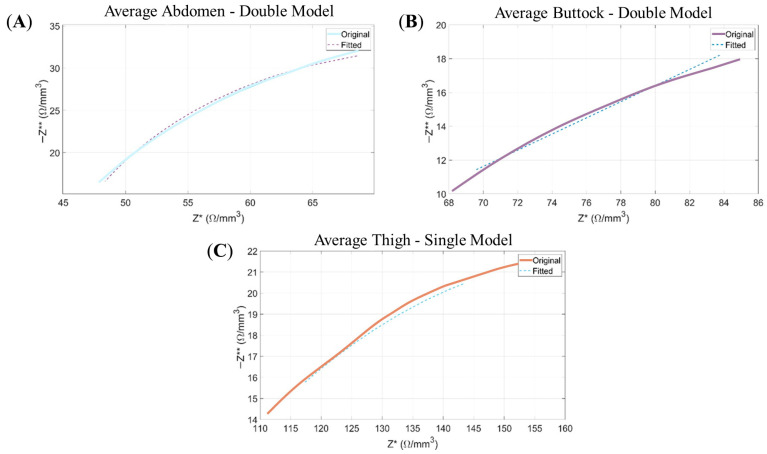
Nyquist plots: Representation of the negative imaginary component of the impedance (−Z**) as a function of its real component (Z*). The continuous line corresponds to the experimental data, while the dashed line illustrates the modelled response obtained from the Double-Dispersion Equivalent Electrical Model. Panels show: (**A**) the average data of abdomen for the Double Model; (**B**) the average data of buttock for Double Model; and (**C**) the average data of thigh for Double Model.

From Figure 6 demonstrates that the Double-Dispersion model achieves a satisfactory fit to the experimental data, as confirmed by the alignment of the Nyquist plots and compliance with the Kramers–Kronig (KK) conditions.

Table 2 presents the quantified values of the equivalent circuit components. Notably, the CPE1-P and CPE2-P parameters approximate unity, suggesting that the Constant Phase Element (CPE) functions analogously to a capacitor.

**Figure 7:**
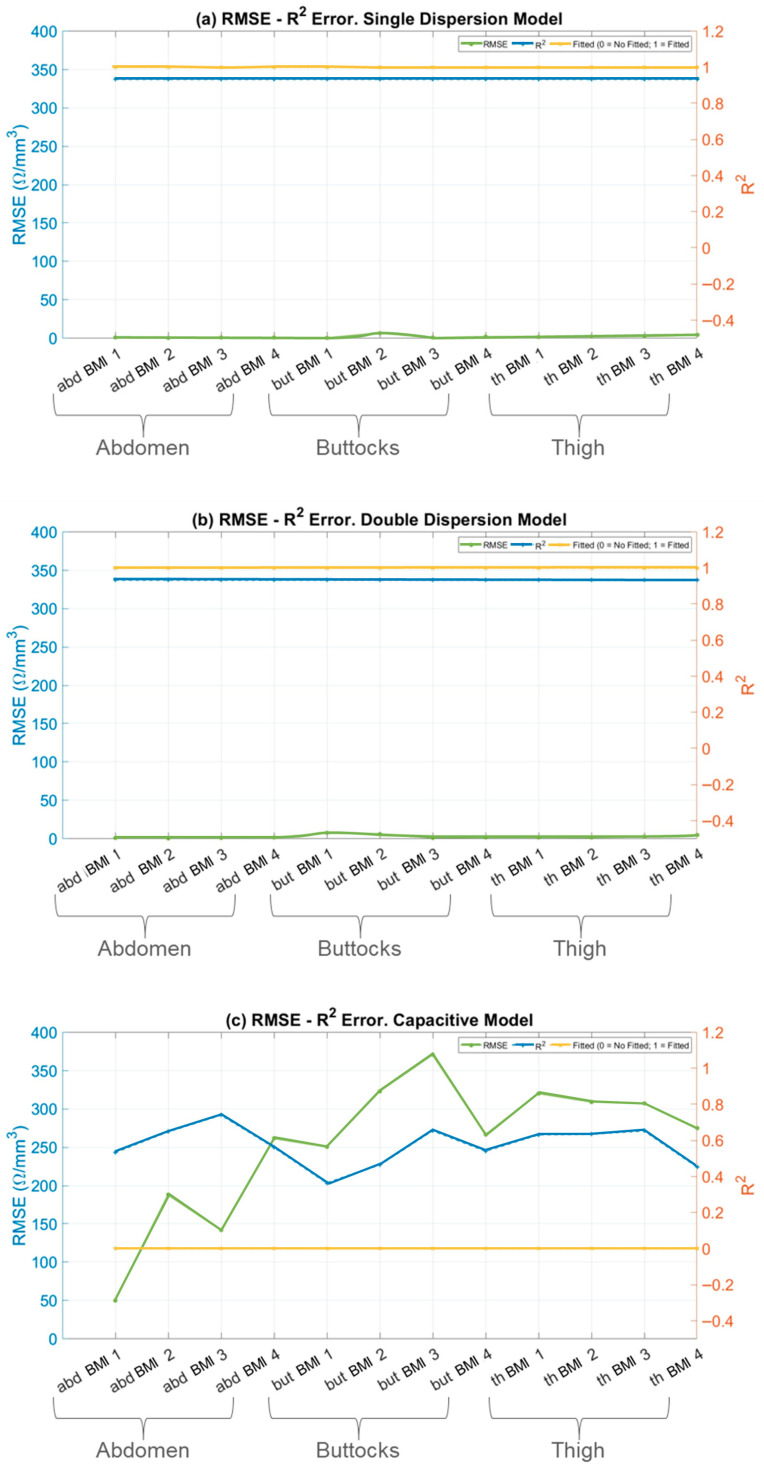

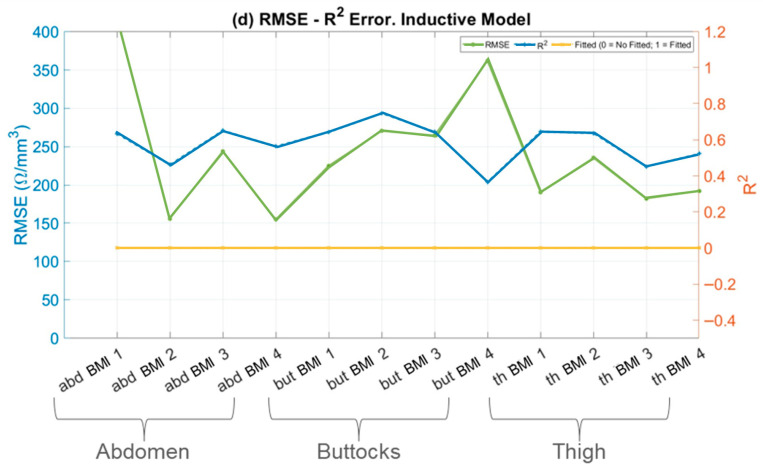
RMSE and R^2^ errors in fitting models. RMSE (left axis, solid line), R^2^ (right axis, dotted line) and status of fitted model (right axis, dotted and dashed line) versus skin fold areas for: (**a**) Single-Dispersion Model; (**b**) Double-Dispersion Model; (**c**) Capacitive Model; and (**d**) Inductive Model. Panels show; abdomen (abd) of BMI 1–4 (Underweight, Normal weight, Overweight and Obesity), buttock (but) of BMI 1–4 (Underweight, Normal weight, Overweight and Obesity), and thigh (th) of BMI 1–4 (Underweight, Normal weight, Overweight and Obesity).

For the capacitive model and for the inductive model, an unacceptable adjustment is observed between the experimental data and the model in terms of the resulting Nyquist plot fit and KK condition. The corresponding results according to this conclusion are presented in Figure 7c,d.

### 3.3. Goodness of Fit Evaluation

For each electrical model and anatomical region, RMSE and R^2^ metrics were evaluated and plotted. Additionally, the anatomical regions were subdivided into four groups based on the subjects’ BMI categories (BMI Scale: Underweight (8 subjects), Normal weight (9 subjects), Overweight (9 subjects), and Obesity (9 subjects). The results are presented below in Figure 7.

In the specific context of skin impedance modelling, the Single-Dispersion Model, Figure 7a, and the Double-Dispersion Model, Figure 7b, exhibit low RMSE values and R^2^ values close to unity. These findings confirm that both the Single- and Double-Dispersion Cole models reliably characterize the impedance behavior of biological tissues across the investigated frequency range. The low RMSE values and high coefficients of determination obtained for both formulations indicate excellent agreement between the measured and modelled impedance spectra, with the Double-Dispersion model consistently providing a marginally improved fit in anatomically heterogeneous regions. Comparable levels of fitting accuracy have been reported in recent bioimpedance studies employing fractional-order Cole models, where low RMSE values and R^2^ coefficients approaching unity were obtained for homogeneous and mildly heterogeneous tissues across similar frequency ranges [17]. The present results extend these observations by demonstrating comparable robustness across multiple adipose tissue regions with distinct anatomical structures.

In contrast, the capacitive model, Figure 7c, and the inductive model, Figure 7d, show poor performance, with significantly higher RMSE values—approximately 100 times higher compared to the well-fitted models. The corresponding R^2^ values for these models are far from unity, indicating that they do not adequately capture the variability and complexity of the skin’s impedance data.

The high RMSE values point to substantial discrepancies between the model predictions and the observed values, while the low R^2^ values reflect the models’ inability to explain most of the variance in the data.

The discrepancy between RMSE and R^2^ highlights the superiority of the Single and Double-Dispersion Cole Models. These models provide a more accurate and complete representation of skin impedance, as they account for both resistive and capacitive components, along with more complex interactions in biological tissues. In contrast, the capacitive and inductive models fail to capture the underlying physical processes governing skin impedance.

While prior works have predominantly focused on single-dispersion formulations or single anatomical sites, the present study demonstrates that a double-dispersion representation provides superior performance when modelling anatomically heterogeneous adipose tissue, particularly in regions such as the abdomen where multilayer structure is more pronounced. This finding supports recent reports suggesting that multiple dispersive processes are required to accurately capture complex tissue architectures [42].

The physical interpretation of the values corresponds to the anatomy of the skin folds. Similarly, the physical interpretation of the fractional—order models also correspond to the anatomy of the skin folds. When comparing the abdomen with the buttocks and legs, it has been observed that the fat cells exhibit different compactness but are divided into two distinct zones below the dermis: the superficial adipose layer and the deep adipose layer, both separated by the fascial plane. Among the different treated zones, the major anatomical difference lies in the superficial layer. In the case of the abdomen, the superficial adipose layer is characterized by non-uniformity and consists of small fat lobules perpendicular to the dermis. Conversely, the layer in the buttock and leg is thinner and exhibits greater homogeneity. These anatomical differences may potentially correlate with the outcomes observed in fractional electrical model settings.

The capacitive and inductive models were not suitable for fitting any of the anatomical regions evaluated. In contrast, both the Single and Double-Dispersion Models demonstrated adequate performance. The Double-Dispersion Model provided a good fit across all three regions—abdomen, buttocks, and thighs—while the Single-Dispersion Model yielded satisfactory results for the buttocks and thighs but failed to adequately model the abdominal region. These differences are attributed to variations in the structure and thickness of the superficial adipose layer.

Accordingly, the Double-Dispersion Model can be interpreted as an electrical analogue of the anatomical configuration of adipose tissue. The two dispersion branches of the model represent the superficial and deep adipose layers, respectively, separated by the fascial plane beneath the dermis. In the abdominal region, the higher heterogeneity and smaller lobule size of the superficial layer correspond to variations in the parameters of the first dispersion branch, while in the buttocks and thighs, the more homogeneous structure of the adipose tissue is reflected in a more stable model response.

These observations reinforce the suitability of the Double-Dispersion Model as the most appropriate framework for accurately describing impedance in all three adipose tissue regions, effectively integrating both electrical and anatomical interpretations.

The reproducibility, selectivity, and detection performance of the extracted CPE-P parameters are summarized in Table 3.

**Figure 8:**
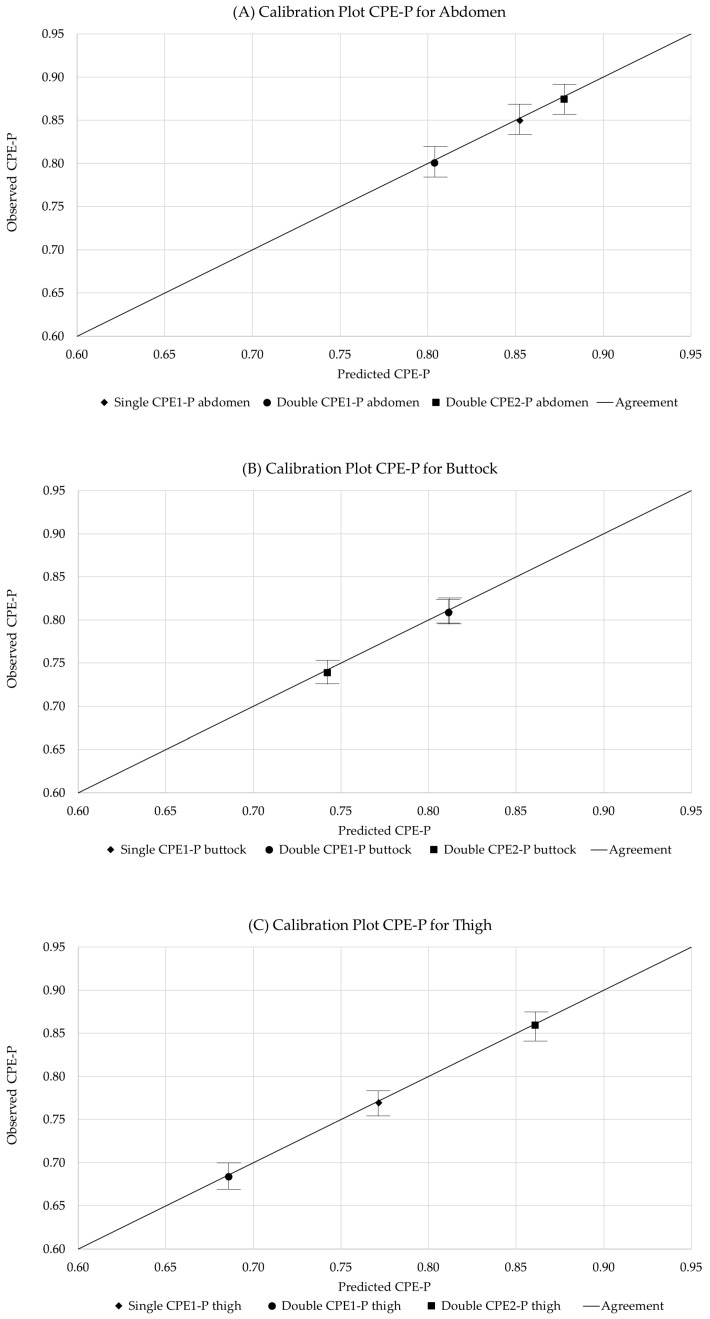
Calibration plot of CPE-P parameters for Single and Double Model, across anatomical regions: (**A**) Abdomen; (**B**) Buttocks; and (**C**) Thigh. In each point the deviation SD of 35 repeated measurements is represented, illustrating reproducibility, selectivity, and the basis for LOD and LOQ estimation. The line indicates the agreement between observed and predicted CPE-P parameters.

The calibration plot for each anatomical section is illustrated in Figure 8.

All parameters exhibited low variability, with coefficients of variation around 3%, demonstrating high reproducibility of both the measurement protocol and model fitting. The observed reproducibility levels (CVav ≈ 3%) are in line with previously reported values for fractional order bioimpedance measurements in soft tissues, where coefficients of variation below 5% are typically considered indicative of robust and repeatable measurement protocols [17,42]. This agreement supports the reliability of the proposed detection framework. Reproducibility was further reflected in the clustering of measurement points around the same predicted values. For example, Single CPE1-P measurements on the abdomen showed tightly grouped points, indicating high reproducibility, whereas Double CPE1-P measurements on the buttock exhibited greater vertical dispersion in Observed CPE-P, reflecting lower reproducibility. Overall, abdomen measurements were more consistent than those on the thigh or buttock.

Selectivity analysis confirmed statistically significant differences between anatomical regions. Single CPE1-P displayed physiologically consistent trends (abdomen > buttock > thigh) and clear discrimination between regions. Double CPE1-P and Double CPE2-P also allowed regional discrimination, although some trends were less consistent. Examination of the calibration plots indicated that abdomen and buttock measurements were better distinguished than abdomen and thigh, with some overlap observed, suggesting moderate overall selectivity.

Limits of detection (LOD) and quantification (LOQ) were estimated based on standard analytical definitions (3σ/S and 10σ/S), as reported in Table 3. For clarity, the LOD and LOQ values presented in Table 3 follow these analytical definitions, whereas the corresponding absolute CPE-P ranges discussed below indicate the practical operating region in which reliable detection and quantification are achieved.

Inspection of the calibration plots (Figure 8) shows that values in the range of 0.6–0.7 Predicted/Observed CPE-P correspond to the lower limit of reliable measurement, with increasing dispersion observed at lower values, indicating higher uncertainty near the LOD. The LOQ was defined as the point at which predicted versus observed values exhibit linearity within a ±10% error margin. Accordingly, the estimated thresholds were LOD ≈ 0.6 CPE-P and LOQ ≈ 0.65–0.7 CPE-P, where data points begin to align closely with the calibration line, confirming the method’s ability to reliably detect and quantify physiologically relevant differences. Among the evaluated parameters, Single CPE1-P provided the most favourable combination of baseline noise, sensitivity, and overall analytical performance.

These findings are consistent with previous reports demonstrating that Cole-based and fractional-order models can reliably describe tissue impedance across different body regions and conditions, while also providing improved fitting accuracy compared to classical integer-order models [17,42]. In contrast to prior studies mainly focused on low-frequency ranges or single-region measurements, our work extends the application of fractional-order models to both abdominal and lower-body adipose tissue and compares single- versus double-dispersion formulations, highlighting the superior performance of the double-dispersion approach. In addition, the explicit evaluation of analytical figures of merit, including reproducibility, selectivity, and detection limits, addresses limitations noted in earlier studies and provides a more complete validation framework for detection-oriented bioimpedance applications.

### 3.4. Anatomical Basis for Electrical Modeling

The selected tissue areas of the body are those characterized by dense fibrous attachments to the underlying deep fascia, which contribute to defining the body’s natural shape and curves. For this study, the abdomen, buttocks, and thighs were chosen [24].

In the abdomen, adipose tissue exhibited a prominent fascial plane running parallel to the dermis, dividing the fat into superficial and deep layers. The superficial adipose layer extended from the subcutaneous fascia to the dermis and was composed of densely packed small fat lobules separated by secondary fibrous septa oriented perpendicular to the skin [29]. The superficial adipose layer of the buttocks and thighs exhibited uniformity throughout the area, showing the narrowest range of individual variation in subcutaneous fat thickness. In the buttocks and thighs, the deep adipose layer appeared dense and compact, characterized by regularly spaced fat lobules bounded by sturdy fibrous septa, often resembling those of the superficial layer.

**Figure 9:**
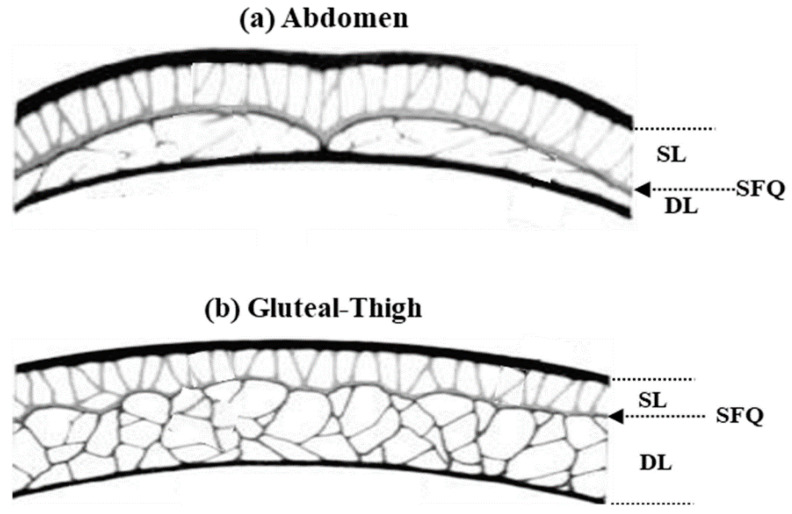
Study of the superficial and deep layers of fat in: (**a**) the abdomen; and (**b**) gluteal and thighs. The panel shows: SL, superficial layer; DL, deep layer; SQF, subcutaneous fat [29].

Figure 9 presents a diagrammatic comparison of subcutaneous adipose tissue in the study areas, highlighting differences in the thickness and architecture of the deep layer [33,38]. It represents the three main differentiated parts of the tissue under study, which could be considered as three serial electrical elements.

The methodology is delineated in two distinguishable parts: First the design of the measurement system and second the processing of data for model fitting. As a proof of concept, for the purposes of this study we prospectively recruited 35 adult human participants for the study, from 1 March until 31 March 2025. The study was conducted in accordance with the Declaration of Helsinki and approved by the Ethics Committee of Hospital Quirón Salud (Barcelona, Spain) under protocol code DEPP-ADIPO-ARW-2024. The data analyzed in this work originates from an ethically approved clinical investigation on non-invasive thermal and cryogenic procedures. Written informed consent was obtained from all participants prior to data acquisition, and all datasets were fully anonymized to ensure participant privacy and confidentiality.

## 4. Conclusions

The results presented in this study demonstrate that fractional-order electrical models effectively characterize the impedance of both abdominal and lower-body adipose tissue, despite their anatomical and structural differences. Among the tested models, the Double-Dispersion Model from the Cole family demonstrates the best fit based on RMSE, R^2^, and KK compliance. This model captures the complex electrical behaviour of skin and adipose tissues with high accuracy, using fewer elements than traditional capacitive or inductive models, which failed to provide adequate fits.

The results confirm that fractional-order bioimpedance models, particularly double-dispersion formulations, provide improved accuracy across different body regions and offer a more comprehensive and robust validation framework than previous approaches.

The fractional-order approach, incorporating constant phase elements (CPEs), enables more realistic modelling of the electrochemical properties of biological tissue. All circuit capacitances were represented using CPEs, providing the flexibility to simulate varying tissue compositions and topologies.

Despite the promising results obtained in this study; several methodological limitations should be acknowledged. The modelling framework assumes tissue homogeneity within each measurement region, which may not fully capture local microstructural variations, particularly in highly heterogeneous adipose tissue. In addition, impedance measurements were performed under baseline environmental conditions; although the models are designed to detect changes associated with thermal or cryogenic processes, dynamic measurements during active temperature modulation were not included in this work.

Reproducibility, while high, may still be influenced by factors such as electrode-tissue contact conditions, hydration level, and minor variations in electrode placement or applied pressure. These sources of variability were mitigated through standardized electrode placement, the use of conductive hydrogel, continuous mechanical stabilization via the suction mechanism, and a rigorous open/short/load calibration procedure that relocates the reference plane close to the tissue interface. As a result, measurement repeatability and reproducibility were preserved across subjects and anatomical regions.

Furthermore, although the selected frequency range does not capture all possible high-frequency dispersive phenomena, it is well suited to characterize the dominant impedance changes associated with adipose tissue freezing and phase transitions, which are the primary focus of this study. Accordingly, while these methodological limitations should be considered when interpreting the results, they do not compromise the reliability or applicability of the proposed fractional-order modelling framework within its intended experimental and clinical context.

The modelling framework supports real-time impedance-based sensing of physiological changes, such as frostbite onset or adipose tissue freezing during aesthetic procedures. These changes alter tissue impedance in measurable ways, enabling the development of closed-loop energy delivery systems with minimal energy consumption and computational cost. As a result, these models not only contribute to safer and more precise treatments but also lay the foundation for low-cost instrumentation in future bio impedance-based medical applications.

The proposed bioimpedance-based method exhibits excellent analytical performance for detection-oriented applications. It demonstrates high reproducibility, moderate but clear selectivity across anatomical regions, and well-defined limits of detection (LOD) and quantification (LOQ). Among the evaluated parameters, Single CPE1-P showed the most robust and consistent analytical behaviour, supporting its selection as the primary sensing variable for quantitative assessment of tissue properties. Reproducibility was highest for abdomen measurements and moderate for thigh and buttock regions, while selectivity was more pronounced between abdomen and buttock than between abdomen and thigh. The method provides reliable measurements above 0.6 CPE-P, with LOQ estimated at 0.65–0.7 CPE-P, confirming its suitability for quantitative analysis within this range.

Overall, the findings validate the Cole-based fractional-order model as a powerful and reliable framework for accurate tissue characterization and monitoring, with potential applications ranging from body contouring and cryotherapy to electrosurgery, cryoablation, and other thermal-based medical interventions. In comparison with prior work, the present study extends the application of fractional-order bioimpedance models to multi-region adipose tissue characterization, demonstrating their suitability for anatomically heterogeneous tissues and detection-oriented applications. Future work will focus on addressing the identified limitations by incorporating dynamic thermal experiments, extended frequency analysis, and subject-specific modelling strategies to further enhance robustness and clinical applicability.

## Figures and Tables

**Table 1:** Adjusted electrical parameter values correspond to the Single-Dispersion Model. CPE1-P corresponds to the CPE exponent P, which is dimensionless. The CPE magnitude (Q) has units F·s^(P−1)^.

Circuit Element Values	Abdomen	Buttock	Thigh
R_1_ (Ω)	74.26	71.63	72.21
R_2_ (Ω)	32.83	33.29	49.63
CPE1-T·10^−9^ (s^CPE-P^/Ω)	5.275	11.837	0.77615
CPE1-P	0.87	0.83	0.77

**Table 2:** Adjusted electrical parameter values correspond to the Double-Dispersion Model. CPE1-P and CPE2-P corresponds to the CPE exponent P, which is dimensionless. The CPE magnitude (Q) has units F·s^(P−1)^.

Circuit Element Values	Abdomen	Buttock	Thigh
R_1_ (Ω)	69.27	67.01	66.55
R_2_ (Ω)	29.71	16.58	39.46
R_3_ (Ω)	9.86	19.40	11.14
CPE1-T·10^−9^ (s^CPE-P^/Ω)	4.013	2.98	0.24
CPE1-P	0.82	0.82	0.68
CPE2-T·10^−9^ (s^CPE-P^/Ω)	281,480.12	17.45	2.93
CPE2-P	0.90	0.78	0.83

**Table 3:** Summary of reproducibility, selectivity, and detection performance of CPE-P parameters (*n* = 35). CVav (%) indicates reproducibility; selectivity shows the mean difference to the closest region. LOD and LOQ were calculated as 3σ/S and 10σ/S, respectively, using baseline SD (abdomen) and minimum inter-regional sensitivity.

Parameter	Region	Mean	SD	CVav (%)	Selectivity (Δ vs. Closest Region)	LOD	LOQ
Single CPE1-P	Abdomen	0.85	0.025	2.97	0.04 (vs. Buttock)	1.9	6.3
	Buttock	0.81	0.024	2.97	0.04 (vs. Abdomen)	—	—
	Thigh	0.77	0.023	2.95	0.04 (vs. Buttock)	—	—
Double CPE1-P	Abdomen	0.80	0.024	2.97	0.01 (vs. Buttock)	7.2	24.0
	Buttock	0.81	0.024	2.95	0.01 (vs. Abdomen)	—	—
	Thigh	0.69	0.020	2.95	0.11 (vs. Abdomen)	—	—
Double CPE2-P	Abdomen	0.88	0.026	2.98	0.02 (vs. Thigh)	3.9	13.0
	Buttock	0.74	0.023	3.07	0.12 (vs. Thigh)	—	—
	Thigh	0.86	0.026	3.02	0.02 (vs. Abdomen)	—	—

## Data Availability

The raw data supporting the conclusions of this article will be made available by the authors on request.
